# Hemoadsorption therapy in severe rhabdomyolysis: a report from the international, prospective COSMOS (CytOSorb^®^ TreatMent of Critically ill PatientS) registry

**DOI:** 10.1080/0886022X.2025.2600720

**Published:** 2025-12-15

**Authors:** Ricard Ferrer, Bartosz Tyczynski, Julian Kreutz, Christina Scharf, Matthias Thielmann, Martin Bellgardt, Andreas Kribben, Philipp Hohlstein, Nuno Germano, Dietrich Henzler, Andreas Baumann, Moritz Unglaube, Ulf Guenther, Weihong Fan, Teresa Klaus, Volker Humbert, Joerg Scheier, Efthymios N. Deliargyris, Fabio Silvio Taccone

**Affiliations:** ^a^ Intensive Care Department, Vall d’Hebron University Hospital, Shock, Organ Dysfunction and Resuscitation Research Group (SODIR), Barcelona, Spain; ^b^ Department of Medical Intensive Care, University Hospital Essen, Essen, Germany; ^c^ Department of Cardiology, Angiology, and Intensive Care Medicine, Philipps University of Marburg, University Hospital, Marburg, Germany; ^d^ Department of Anesthesiology, LMU university hospital, LMU Munich, Munich, Germany; ^e^ Department of Thoracic and Cardiovascular Surgery, West German Heart & Vascular Center Essen, University Hospital Duisburg-Essen, Essen, Germany; ^f^ Department of Anesthesiology and Intensive Care Medicine, St Josef-Hospital Bochum, Bochum, Germany; ^g^ Department of Nephrology, University Duisburg-Essen, University-Hospital Essen, Essen, Germany; ^h^ Department for Gastroenterology, Metabolic Disorders and Intensive Care Medicine (Department of Medicine III), RWTH-University Hospital Aachen, Aachen, Germany; ^i^ Emergency Department, Hospital Curry Cabral, Lisboa, Portugal; ^j^ Department of Anesthesiology, Surgical Intensive care, Emergency and Pain Medicine, Ruhr-University Bochum, Hospital Herford, Herford, Germany; ^k^ Department of Anesthesiology, Intensive Care Medicine and Pain Management, BG University Hospital Bergmannsheil, Medical Faculty of Ruhr University Bochum, Bochum, Germany; ^l^ Department of Intensive Care Medicine, Helios Dr. Horst- Schmidt Klinik Wiesbaden, Wiesbaden, Germany; ^m^ Hospital Oldenburg AöR, University Hospital of Anesthesiology and Intensive Care, Oldenburg, Germany; ^n^ Medical Affairs, CytoSorbents Corporation and CytoSorbents Medical Inc, Princeton, New Jersey, USA; ^o^ Medical Affairs, CytoSorbents Europe GmbH, Berlin, Germany; ^p^ Department of Intensive Care, Hôpital Universitaire de Bruxelles (HUB), Université Libre de Bruxelles (ULB), Brussels, Belgium

**Keywords:** CytoSorb, hemoadsorption, blood purification, rhabdomyolysis, registry, AKI

## Abstract

Severe rhabdomyolysis is associated with an increased risk of acute kidney injury (AKI) and mortality. Hemoadsorption may reduce myoglobin levels and mitigate AKI. The COSMOS registry is a prospective, multicenter, observational study evaluating hemoadsorption with CytoSorb^®^ (CS) in critically ill patients. Consecutive eligible patients are enrolled without preselection, and data collected at multiple timepoints: 24 h before CS; during CS and 24 h post-treatment; at ICU and at hospital discharge and at 90-day follow-up. The current analysis examined the effects of hemoadsorption in patients with severe rhabdomyolysis (e.g. myoglobin > 10,000 µg/L) by comparing changes in myoglobin and creatinine levels over time and the associated clinical impact. A total of 31 patients (mean age 55 years; 19% female) were included; CS was either integrated into renal replacement therapy (RRT) (*n* = 25, 81%) or used in stand-alone hemoperfusion mode (*n* = 6, 19%). When comparing pre- to post- CS treatment, significant reductions in mean myoglobin (24,109 ± 28,777 to 4,251 ± 4,399 µg/L; *p* = 0.001) and creatinine levels (3.5 ± 2.9 to 2.4 ± 1.5 mg/dL; *p* = 0.013) were observed. ICU mortality occurred in 8 (26%) patients. All survivors (*n* = 23) were able to be discharged from ICU without renal support. During the 90-day follow-up only one of these patients required dialysis again. Furthermore, there were no serious device-related adverse effects reported. The current study suggests that hemoadsorption is an effective method to rapidly and safely reduce myoglobin levels in severe rhabdomyolysis and may improve kidney function. These encouraging results will require validation in larger, controlled studies.

## Graphical Abstract

**Figure:**
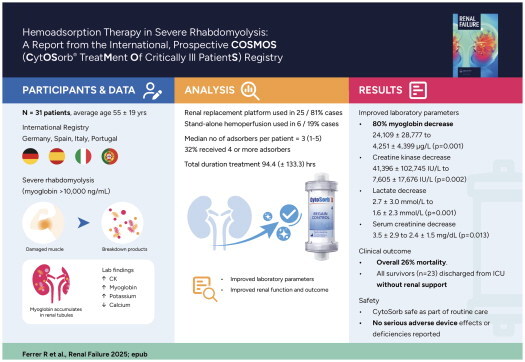

## Background

Rhabdomyolysis is a clinical syndrome characterized by the breakdown of muscle tissue, leading to the release of myoglobin into the bloodstream [1]. Excessive circulating myoglobin can lead to its accumulation and precipitation into the renal tubules, formation of intratubular casts and direct toxicity to tubular cells, impairing kidney function [2]. Inflammation, further amplified by the precipitating muscle injury, exacerbates this pathological process. The combined effects of myoglobin-induced toxicity and inflammation can lead to acute tubular necrosis, a common cause of acute kidney injury (AKI), which is often associated with electrolyte imbalances, acidemia, organ dysfunction and increased systemic inflammatory response [3]. High myoglobin levels are also associated with increased risk of mortality, making early recognition and management of myoglobinuria and inflammation crucial in patients with muscle injury [4]. Treatment primarily focuses on supportive care, including aggressive fluid resuscitation, organ support and management of potential complications [5]. However, in cases of severe rhabdomyolysis with concomitant multi-organ failure or refractory hemodynamic instability, additional therapeutic interventions may be indicated.

CytoSorb^®^ (CS), is a CE-marked blood purification technology for the removal of cytokines, bilirubin and myoglobin, and may therefore be a valuable treatment option in patients with severe rhabdomyolysis; CS works by primarily removing hydrophobic substances from the bloodstream *via* hemoadsorption. Such substances may be involved in the pathophysiology of many critical conditions, such as sepsis, trauma, rhabdomyolysis and others. A recent consensus statement of international experts concluded that adjuvant hemoadsorption therapy in severe rhabdomyolysis is both feasible and safe and may be an effective method to reduce elevated circulating levels of myoglobin [6]. Early clinical reports have suggested that rapid myoglobin reductions by CS may translate into renal function recovery [7]. However, it remains unclear whether early removal of elevated myoglobin levels, even before the initiation of renal replacement therapy (RRT), can prevent the development of AKI. Additionally, the long-term impact on renal function when CS is used in combination with RRT requires further investigation. The International COSMOS (CytOSorb TreatMent Of Critically Ill PatientS) Registry, an ongoing, multi-center, real-world data collection initiative, aims to evaluate the clinical performance of CS in critically ill patients, including those with rhabdomyolysis [8]. This registry includes data from diverse clinical settings, offering insights into the use of CS as part of standard care. In the current analysis, we analyzed CS use in patients with severe rhabdomyolysis, focusing on its impact on key laboratory and clinical parameters such as myoglobin levels, renal function, hemodynamic stability, and overall survival.

## Methods

### Registry study design

The COSMOS Registry is a prospective, multicenter, international observational study conducted in countries where CS is approved and routinely used in everyday practice [8,9]. The registry began enrollment on July 15, 2022, and is currently active in Germany, Spain, Italy, Portugal, Austria and Poland with the intent of additional geographic expansion in the future. Participating sites are selected based on their routine use of CS, expected patient enrollment, clinical study expertise, data collection capabilities, and interest in participation. Training is provided to all site personnel, including investigators and coordinators, to ensure proper device use according to the approved instructions for use and adherence to protocol procedures. Consecutive patients meeting eligibility criteria are enrolled and treated according to each participating site’s routine standard of care. No specific treatment guidelines for patient management are included in the protocol, except for the requirement for appropriate device use according to available best practice recommendations and data collection at the specified timepoints. Following IRB/IEC approval, written informed consent is obtained from all participants or their legal representatives before any data collection.

### Patient population, data collection, and outcomes

Data were systematically collected at multiple timepoints, including at baseline and within 24 h prior to CS initiation, during CS treatment, and 24 h post-CS treatment, as well as at ICU discharge, hospital discharge, and a final follow-up on day 90. Vital status is also evaluated at the same timepoints. For the current analysis, the primary outcome was change in myoglobin levels over the course of treatment with CS. Secondary outcomes included: creatine kinase (CK) and creatinine levels, vasopressor requirements and doses and changes in daily fluid balance, PaO_2_/FiO_2_ ratio, kidney function on day 90 and survival status. Device safety was assessed *via* investigator-reported serious adverse device-related events (SADEs) or Device Deficiencies (DD), up to ICU discharge or death, whichever occurs first.

### Statistical analysis

A pre-specified statistical analysis plan was adhered to for summarizing and analyzing the data variables. For continuous variables, either median with interquartile range (IQR) or mean with standard deviation are presented. Categorical variables were summarized by counts and proportions for each category. Baseline data were captured as the time of ICU admission for demographic information, medical history, comorbidities, and standardized risk score calculations. The pre-CS therapy period was defined as the 24 h before the start of CS for assessing vasopressors, fluid balance, and the most recent laboratory and blood gas values. The post-CS therapy period was defined as the 24 h following the end of CS therapy for vasopressor and fluid balance data, and the earliest available values after CS therapy cessation for laboratory tests and blood gases. Absolute changes from pre-CS therapy were calculated by subtracting pre-CS from post-CS values. The Wilcoxon signed-rank test was used to compare paired data (pre- and post-CS therapy) and to assess changes associated with CS treatment. All p-values were rounded to a maximum of four decimal points. All statistical analyses were conducted using SAS 9.4 (SAS, Cary, NC).

## Results

### Patient characteristics

The current analysis includes 31 patients with severe rhabdomyolysis. Demographics of the study population are shown in Table 1. Importantly, median baseline Acute Physiology and Chronic Health Evaluation (APACHE) II score on admission was 26, indicating a predicted in-hospital mortality of 40-50%.

**Table 1.** Baseline characteristics of the first 31 patients in the COSMOS registry (*n* and percent shown for categorial variables, median (IQR) for continuous variables except age and body weight which are presented as mean and standard deviation).

Baseline characteristics	*n* = 31
Age (years), mean ± SD	55 ± 19
Age ≥65	11/31 (35.5%)
Age <65	20/31 (64.5%)
Gender, *n* (%)	
Male	25/31 (80.6%)
Female	6/31 (19.4%)
Weight (kg), mean ± SD	94 ± 27
Charlson comorbidity score, median (IQR)	3 (1,5)
APACHE II score, median (IQR)	26 (20, 32)

APACHE: Acute physiology and chronic health evaluation; SD: Standard deviation; IQR: Interquartile range, N: Number.

### Treatment modalities of CytoSorb^®^ therapy

**Figure 1.**
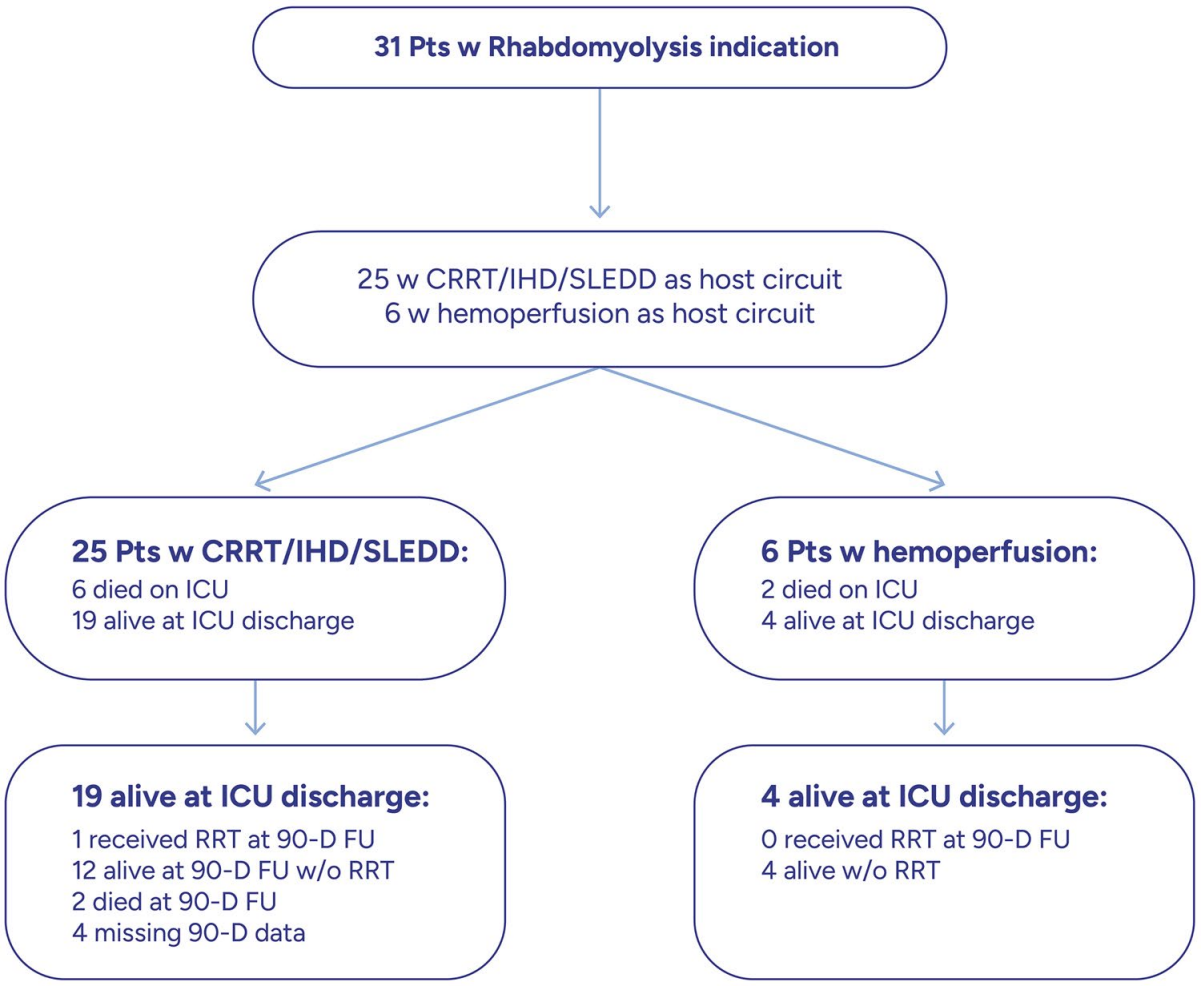
Flowchart of patient numbers until 90-day follow-up; pts: patients; (C)RRT: (Continuous) renal replacement therapy; IHD: intermittent hemodialysis; SLEDD: Sustained Low-Efficiency Daily Dialysis; ICU: intensive Care Unit; 90-D FU: 90 days Follow-up.

The host circuit used for integration of CS was renal replacement therapy (RRT) in the majority of cases (25/31, 81%), either run as continuous renal replacement therapy (CRRT; 71%) or intermittent hemodialysis/sustained low-efficiency daily dialysis (IHD/SLEDD; 10%). Stand-alone hemoperfusion was used in the remaining 6 patients (Figure 1). The median number of CS adsorbers used per patient was 3 (1–5), with 10 (32%) patients having received 4 adsorbers or more. Mean daily duration of adsorber usage was 18.2 (± 9.9) hours, with the mean total duration of the overall treatment being 94.4 (± 133.3) hours.

### Effects on laboratory and hemodynamic parameters

**Figure 2.**
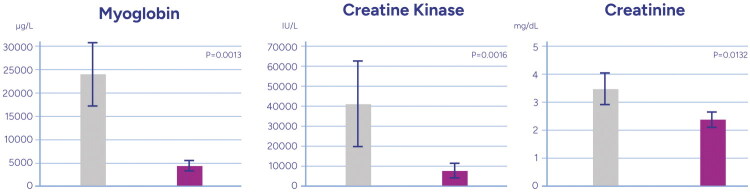
Changes in myoglobin, creatine kinase, creatinine in 24 h periods before (grey) versus after CS treatment (lilac) in the entire cohort. The mean ± standard error bar is presented for lab change. The Wilcoxon signed rank test was performed to test for the null hypothesis that the median of changes is equal to 0.

Compared to baseline values, significant reductions in relevant laboratory parameters were noted after CS therapy (Figure 2, Table 2). Specifically, myoglobin concentrations decreased from 24,109 ± 28,777 to 4,251 ± 4,399 µg/L (*p* = 0.001); CK levels decreased from 41,396 ± 102,745 IU/L to 7,605 ± 17,676 IU/L (*p* = 0.002); lactate levels decreased from 2.7 ± 3.0 to 1.6 ± 2.3 mmol/L (*p* = 0.001). CS therapy was also associated with a significant decrease in serum creatinine, from 3.5 ± 2.9 to 2.4 ± 1.5 mg/dL (*p* = 0.013).

**Table 2.** Change of laboratory and hemodynamic parameters from baseline before CS therapy versus post-CS therapy; CS: CytoSorb; RRT, renal replacement therapy; PaO2/FiO2, partial pressure of oxygen to fraction of inspired oxygen ratio.

Parameter	Baseline value (Mean ± SD)	Post-CS therapy (Mean ± SD)	*p*-value
Myoglobin (µg/L)	24,109 ± 28,777	4,251 ± 4,399	0.001
Creatinine kinase (IU/L)	41,396 ± 102,745	7,605 ± 17,676	0.002
Lactate (mmol/L)	2.7 ± 3.0	1.6 ± 2.3	0.001
Serum creatinine (mg/dL)	3.5 ± 2.9	2.4 ± 1.5	0.013
Fluid balance (mL, 24h pre vs. post CS)	+1,716 ± 1,776	520 ± 2,834	0.030
Norepinephrine dosage (µg/kg/min)	0.24	0.16	0.07
Norepinephrine dosage in ICU survivors (µg/kg/min)	0.16 ± 0.14	0.08 ± 0.07	0.035
PaO2/FiO2 ratio (mmHg)	193 ± 82	218 ± 111	0.233

Of note, a reduction in creatinine of a similar magnitude as that seen in the overall cohort, was also observed among the 6 patients where CS was used without concomitant RRT in stand-alone hemoperfusion mode (3.1 ± 2.0 to 2.1 ± 1.2 mg/dL). The use of CS was associated with a significant reduction in fluid balance (+1,716 ± 1,776 mL in the 24 h before CS treatment versus 520 ± 2,834 mL in the 24 h post-CS treatment; *p* = 0.030). Interestingly changes in norepinephrine dosage in the overall cohort were not significant (from 0.24 to 0.16 µg/kg/min, *p* = 0.07), however, they were significant among ICU survivors (from 0.16 ± 0.14 µg/kg/min to 0.08 ± 0.07 µg/kg/min - *p* = 0.035). Oxygenation remained stable during CS treatment, with a PaO_2_/FiO_2_ ratio of 193 ± 82 mmHg at baseline and 218 ± 111 mmHg at the end of CS therapy (*p* = 0.233).

**Figure 3.**
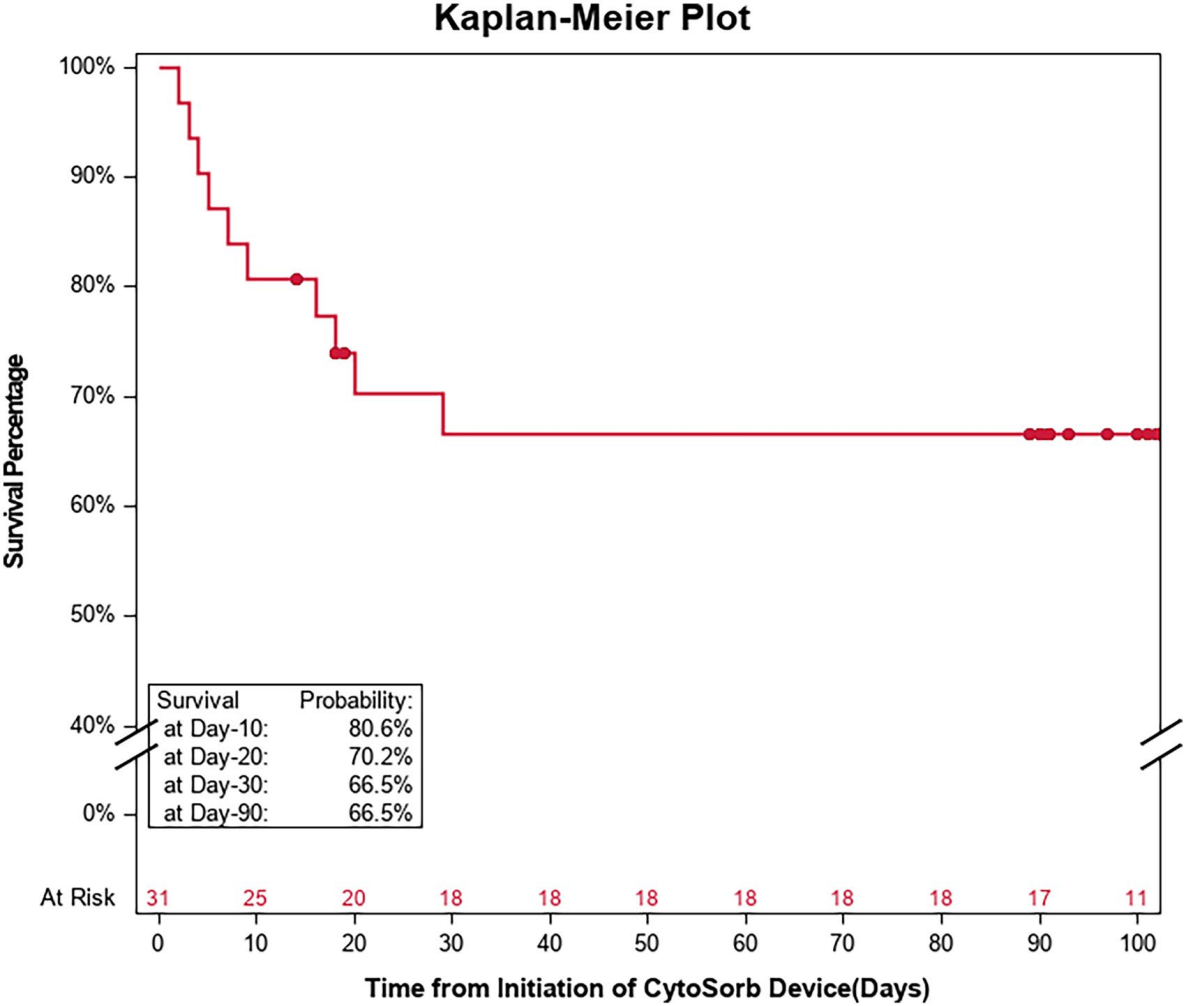
Kaplan–Meier curve for survival of patients until day 90 post CytoSorb initiation.

**Figure 4.**
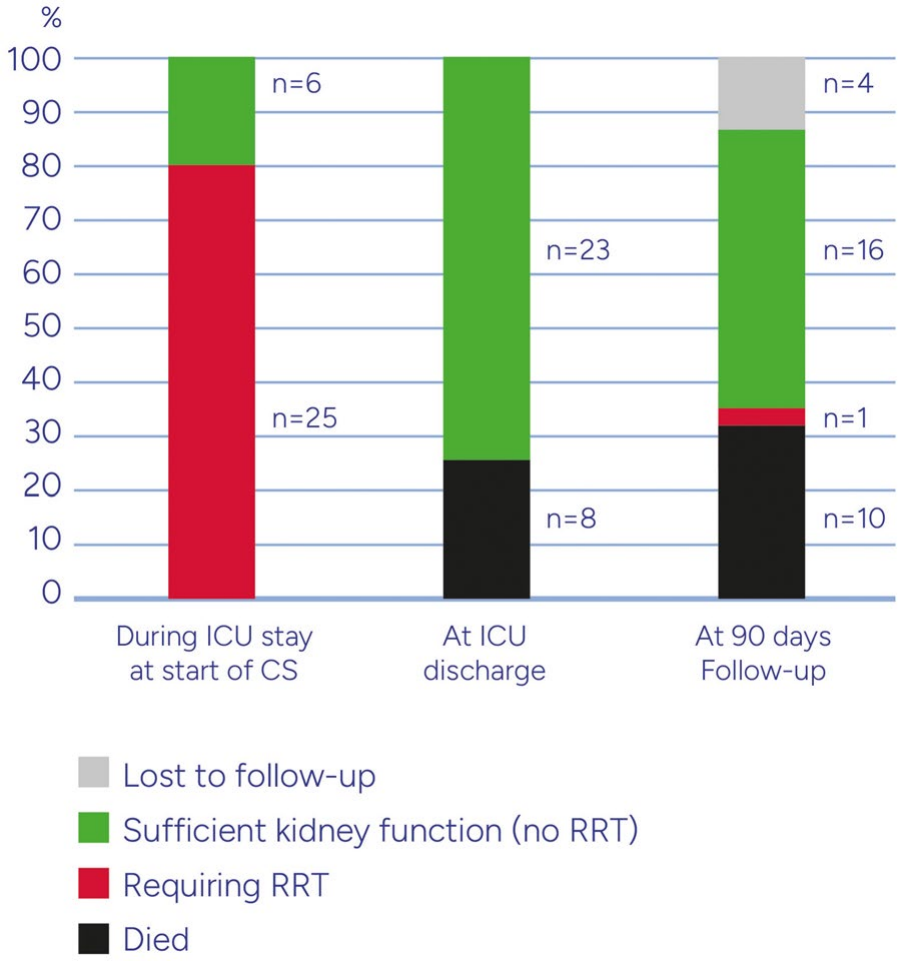
Kidney function outcome of all patients during ICU and start of CS, at ICU discharge and at 90 days follow up.

**Figure 5.**
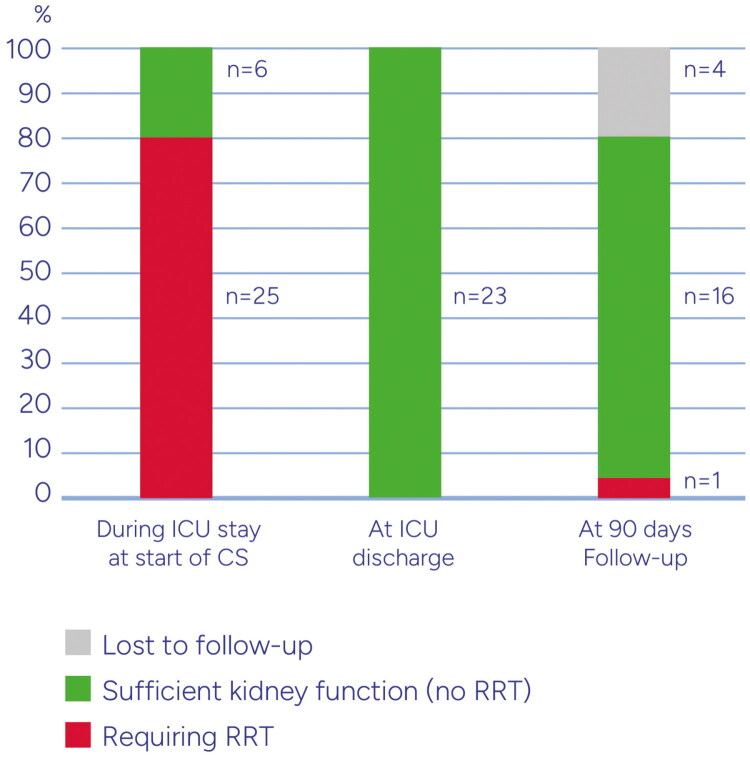
Kidney function outcome of surviving patients during ICU and start of CS, at ICU discharge and at 90 days follow up.

Figure 3 shows the Kaplan Meier plot for survival of patients up to day 90. Excluding patients who died in the ICU as well as the ones with missing data on RRT stop day or ICU discharge day, the median dialysis-free days before ICU discharge was 3.5 [2, 9] days. Survival and requirement for organ support while in ICU as well as during the 90-day follow-up are depicted in Figures 4 and 5. Six patients from the RRT+CS group and 2 from the hemoperfusion + CS group died during their ICU stay. Among the survivors, only one patient who had required RRT during ICU stay, required dialysis again. The median length of ICU stay was 15 (10–26) days and ICU mortality occurred in 8 (26%) patients.

### Safety

There were no serious adverse device effects or device deficiencies reported. Also, pre-CS to post-CS platelet counts (158 ± 88 × 109/L vs. 132 ± 90 × 109/L; *p* = 0.21) and albumin (2.7 ± 0.5 vs. 2.5 ± 0.4 g/dL; *p* = 0.12) remained stable during CS therapy.

## Discussion

The current analysis of the international, prospective COSMOS registry reports outcomes of consecutive patients with severe rhabdomyolysis treated with CS hemoadsorption as part of their routine care. There are three main observations on the impact of CS therapy derived from this analysis. First, significant reductions were observed on relevant laboratory parameters including myoglobin, creatine kinase, creatinine and lactate. Second, most patients with an initial need for RRT showed recovery of kidney function upon ICU discharge or remained dialysis-free when hemoadsorption was used prior to the development of AKI. Finally, CS use as part of routine care in patients with severe rhabdomyolysis was safe without any serious device related adverse events reported.

Elevated levels of myoglobin contribute to the development of AKI *via* direct and indirect mechanisms, as recently agreed in the consensus of the Hemoadsorption in Rhabdomyolysis Task Force [6]. This group also stated that patients with elevated myoglobin levels of >10,000 µg/L should be considered as a high risk-subgroup and myoglobin removal as an appropriate approach. Furthermore, hemoadsorption with CS was seen to be effective in this regard, while conventional RRT did not significantly eliminate myoglobin from the blood. These consensus statements are based on analysis by Scharf et al. [10], who showed significant reductions in myoglobin levels with the adsorber in patients with severe rhabdomyolysis. Further studies have confirmed these findings [11–13] and the results from the present analysis also strongly support the principal efficacy of CS hemoadsorption in reducing elevated levels of myoglobin.

In line with recommendations from the consensus task force, in the current cohort, baseline levels of myoglobin prior to initiation of hemoadsorption were highly elevated (>10,000 µg/L) and could be reduced in a clinically relevant and statistically significant manner down to levels <5,000 µg/L, which is seen as a potential criterion to consider ending hemoadsorption [6]. Our findings support the >10,000 µg/L myoglobin threshold as a practical trigger for considering early hemoadsorption, while emphasizing that larger studies and individualized clinical judgment remain essential.

Potential saturation of the adsorber has been discussed in some publications leading to the recommendation to renew the cartridge after 8–12 h, if further myoglobin reduction is needed [12]. The increased duration between adsorber changes of 18 h in this cohort might reflect hurdles in the practical realization of the expert recommendations in everyday practice. Generally, an individualized treatment approach and absorber change schedule based on the stage of development of the disease process and circulating myoglobin and CK levels is warranted. For example, ongoing rhabdomyolysis, indicated by rising CK levels will likely require more adsorbers than stable/resolving rhabdomyolysis with ongoing reductions in CK.

Direct removal of CK (around 80 kDa) by the adsorber is not expected due to the molecular weight that exceeds size-selective removal cutoff of the device (up to 60 kDa) [12].

Based on pathophysiological considerations, reductions in circulating levels of myoglobin may translate into benefits in renal function [6]. This is supported by a recent propensity score matched analysis of patients with severe rhabdomyolysis requiring renal replacement therapy, that showed marked improvements in renal recovery after 30 days in the patient group who additionally received CS therapy. Considering patients who survived 30 days, nearly 3 times as many patients no longer needed renal replacement therapy in the CS treated group compared to the RRT only group, with a mean risk reduction of nearly 40% and a number needed to treat of 3 [7]. Improved kidney function under hemoadsorption therapy has also been observed in the setting of cardiac surgery during use in orthotopic heart transplantation [14].

The present results show a significant decrease in creatinine when hemoadsorption was combined with RRT. While it is logical to postulate that this reduction is primarily attributable to by renal replacement therapy, a decrease of similar magnitude was also observed in patients who received CS therapy *via* the stand-alone hemoperfusion mode and without RRT. Important support for the meaningful benefits in renal function is the fact that all 19 patients who survived ICU and had required RRT during their stay there, no longer needed RRT on ICU discharge. Importantly, during the 90-day follow-up, only one of these patients required RRT again (see Figures 3 and 4). These high numbers of renal recovery associated with the use of CS are encouraging and exceed those from a recent analysis [7].

Another approach for hemoadsorption in this setting might be to try to prevent AKI instead of treating it, as also described in some case reports [15,16]. This may also have been the target in the six patients who did not have severe kidney dysfunction at initiation of CS, which was then consequently used in stand-alone mode. Apart from two patients who died in the ICU, the four other stand-alone patients remained dialysis-free after 90 days. A recent animal study in the setting of sepsis-associated AKI investigated the effects of CS hemoadsorption on kidney oxygenation and perfusion and showed an improvement in multiple determinants in the CS treated animals [17]. While this was a sepsis and not a rhabdomyolysis model, inflammation is also likely to contribute to rhabdomyolysis-associated AKI, and these findings might further support the potential benefits of hemoadsorption in the setting of rhabdomyolysis.

Hemoadsorption has also been discussed as potentially exerting protective effects on the endothelial function in hyperinflammatory conditions [14], which might help explain the beneficial effect on fluid balance. This is critically important and has been previously reported in patients with septic shock [18]. Although fluid management in rhabdomyolysis is used to prevent AKI, in septic shock it is used for resuscitation and improving oxygen delivery, the ability to achieve the desired treatment objectives while preventing fluid overload is probably beneficial in both conditions.

The current results support several key statements from the “Hemoadsorption in Rhabdomyolysis Task Force” Consensus and validate CS as an effective therapeutic option for severe rhabdomyolysis. The following Table 3 compares findings of this study to recommendations of the Rhabdomyolysis Task Force to benchmark real-world registry experience against published recommendations.

**Table 3.** Alignment of hemoadsorption in rhabdomyolysis task force consensus [6] with COSMOS registry findings.

Consensus recommendation	COSMOS registry findings
Who to treat: Consider extracorporeal removal when myoglobin >10,000 µg/L; prioritize myoglobin over CK.	Most treated patients had very high myoglobin; effects were strongest in those with extreme baselines, consistent with high-risk subgroup.
When to start: Initiate within 24 h of severe rhabdomyolysis, ideally with CRRT if renal indications exist.	Early initiation feasible; often preceded need for dialysis, supporting a prevention-oriented approach.
Modality choices beyond CRRT: Stand-alone hemoperfusion when CRRT not required; integrate with CRRT when indicated.	Both pathways used; stand-alone adsorption achieved creatinine improvements similar to overall cohort, showing effectiveness beyond CRRT.
If myoglobin unavailable: Proceed based on clinical picture and elevated CK.	Myoglobin as well as CK were measured by the contributing sites regularly. However direct impact of the different laboratory parameters on indication and decision making remains unclear in the registry data.
Cartridge management: Exchange every 8–12 h until myoglobin <10,000 µg/L; check for rebound and re-install if needed.	Adsorber interval changes were markedly longer in the registry with a mean of 18.2 h reflecting the challenges of putting theoretical considerations into everyday clinical practice
When to stop in AKI: Adsorption may be stopped before CRRT ends; continue until myoglobin consistently <5,000 µg/L.	Levels of mean myoglobin after CS Therapy below 5,000 µg/L reflect this recommendation.
Clinical trajectory/outcomes: Case series suggest renal recovery; evidence quality low, trials encouraged.	Larger, structured cohort with high renal recovery and RRT avoidance among survivors, strengthening practice-based evidence.
Safety: No severe device-related adverse events; rare mild thrombocytopenia reported.	No major device-related complications observed, confirming safety and feasibility.
Systems integration/multi-organ support: Adsorption can be embedded into CRRT, intermittent dialysis, ECMO, or bypass circuits.	Applied across stand-alone, intermittent dialysis, and CRRT; supports flexible integration and multi-organ support strategies.

Furthermore, we show that stand-alone CS in hemoperfusion mode (without CRRT) was feasible and successfully applied in about 20% of the included subjects in the current analysis. Current prevention strategies for rhabdomyolysis-associated AKI emphasize early recognition of high-risk patients (e.g. myoglobin >10,000 µg/L), aggressive yet judicious fluid resuscitation, hemodynamic optimization, avoidance of nephrotoxins, and close laboratory monitoring [18]. Within this framework, rapid reduction of circulating myoglobin may interrupt pigment-nephropathy before established tubular injury. In our cohort, the pattern of rapid myoglobin reduction, improvement in creatinine (including those patients without concomitant RRT), and high dialysis independence among survivors aligns with a prevention-oriented strategy: deploy adsorption early when biochemical thresholds are high or increasing, then individualize cartridge-exchange intervals based on CK/myoglobin kinetics and clinical course. Our results therefore extend the practical scope of extracorporeal care from sole rescue therapy for established AKI to more risk-adapted prevention in patients at imminent risk.

Some approaches presented in our registry also align with the idea of biomarker-guided AKI management. Early markers, either functional ones like creatinine and urine output, or biochemical ones such as very high myoglobin, can signal risk before full renal failure is established [19]. Using hemoadsorption in a stand-alone set-up at this stage would mean reacting to a strong laboratory signal with an extracorporeal intervention.

Other approaches follow the idea of “damage control nephrology”, where the focus is not to wait until severe AKI is established but to contain the insult early [20]. By lowering circulating myoglobin and limiting tubular exposure, adsorption works alongside conservative measures such as fluid optimization, alkalinization, and avoiding nephrotoxins. In this way it can help slow down or prevent progression when standard measures are not enough.

Finally, our experience shows that extracorporeal adsorption should not be seen as only a nephrology tool but as part of multi-organ support. Adsorption can be connected not just to CRRT or standalone hemoperfusion, but also to ECMO, cardiopulmonary bypass, or intermittent dialysis [21]. This makes it possible to use the technique in different extracorporeal circuits that are already running in critical care. Such an approach fits with current practice in intensive care, where organ support strategies are increasingly combined rather than handled separately. In this sense, adsorption can act as a link between protecting the kidney and stabilizing the whole patient.

Importantly, no Serious Adverse Device Effects (SADE) or Device Deficiencies (DDs) were reported. In aggregate, these observations support the consideration of CS hemoadsorption in the management of severe rhabdomyolysis, particularly in patients with very high myoglobin levels (>10,000 µg/L) who are at imminent risk or already have evidence of AKI. Although there have been no serious device-related adverse events reported in the COSMOS registry to date, it is important to highlight potential risks associated with the clinical use of the device. First, the extracorporeal nature of the device raises the possibility of unintended removal of concomitant medications [22]. Additionally, a transient decrease in platelet count has been observed in some patients. While these findings have not translated into serious adverse events including bleeding events in the registry, they underscore the importance of ongoing monitoring and risk mitigation.

### Limitations

The major limitation of this observational registry is the lack of a concurrent control group and therefore definitive inference cannot be made for the association of CS therapy with the clinical outcomes reported. As patient numbers increase, we plan to investigate the potential of performing a comparative analyses with suitable synthetic control arms of propensity matched subjects not treated with the device. A second limitation is related to the lack of detailed information on the underlying cause of rhabdomyolysis (e.g. trauma, drug-induced, or other etiologies) as well as detailed assessment of kidney function by the standardized KDIGO definition. Finally, the inability to clearly distinguish whether RRT was initiated as a direct clinical necessity for AKI or primarily to facilitate hemoadsorption therapy is another noteworthy limitation. The registry does not specify or capture the specific clinical indications for initiation of RRT or CytoSorb therapy, which introduces center-level variability in treatment intent.

## Conclusions

In the current analysis of the COSMOS registry, CS therapy rapidly improved myoglobin levels and kidney function in patients with severe rhabdomyolysis. Long term follow-up revealed a high rate of patients no longer requiring renal replacement therapy. CS therapy was also associated with improved fluid balance and no serious adverse device effects were reported.

## Data Availability

The data are available upon reasonable written request and with written permission of the CytoSorbents Corporation. Data Management Plan and Informed Consent Forms are available upon request.

## Abbreviations

90-D FU: 90 days follow-up
APACHE: Acute physiology and chronic health evaluation
AKI: Acute kidney injury
(C)RRT: (Continuous) Renal replacement therapy
CS: CytoSorb^®^
DD: Device deficiencies
FiO2: Fraction of inspiratory oxygen concentration
ICF: Informed consent form
ICH: International conference on harmonization
ICU: Intensive care unit
IHD: Intermittent hemodialysis
IEC: Independent ethics committee
ISO: International organization for standardization
IQR: Interquartile range
KDIGO: Kidney disease: improving global outcomes
NE: Norepinephrine
PaO2: Partial pressure of oxygen
P/F ratio: Ratio of partial pressure of oxygen in arterial blood (PaO2) to the fraction of inspiratory oxygen concentration (FiO2)
RCT: Randomized control trial
SD: Standard deviation
SLEDD: Sustained low-efficiency daily dialysis
USA: United States of America
